# Icebergs, sea ice, blue carbon and Antarctic climate feedbacks

**DOI:** 10.1098/rsta.2017.0176

**Published:** 2018-05-14

**Authors:** David K. A. Barnes, Andrew Fleming, Chester J. Sands, Maria Liliana Quartino, Dolores Deregibus

**Affiliations:** 1 British Antarctic Survey, Natural Environment Research Council, Madingley Road, Cambridge CB3 0ET, UK; 2 Departamento de Biología Costera, Instituto Antártico Argentino, 25 de Mayo 1147 (PC 1650), San Martín, Buenos Aires, Argentina; 3 Museo Argentino de Ciencias Naturales ‘B. Rivadavia’. Av. A. Gallardo 470 (C1405DJR), Buenos Aires, Argentina

**Keywords:** blue carbon sink, Southern Ocean, iceberg A68, climate change, benthos, phytoplankton

## Abstract

Sea ice, including icebergs, has a complex relationship with the carbon held within animals (blue carbon) in the polar regions. Sea-ice losses around West Antarctica's continental shelf generate longer phytoplankton blooms but also make it a hotspot for coastal iceberg disturbance. This matters because in polar regions ice scour limits blue carbon storage ecosystem services, which work as a powerful negative feedback on climate change (less sea ice increases phytoplankton blooms, benthic growth, seabed carbon and sequestration). This resets benthic biota succession (maintaining regional biodiversity) and also fertilizes the ocean with nutrients, generating phytoplankton blooms, which cascade carbon capture into seabed storage and burial by benthos. Small icebergs scour coastal shallows, whereas giant icebergs ground deeper, offshore. Significant benthic communities establish where ice shelves have disintegrated (giant icebergs calving), and rapidly grow to accumulate blue carbon storage. When 5000 km^2^ giant icebergs calve, we estimate that they generate approximately 10^6^ tonnes of immobilized zoobenthic carbon per year (t C yr^−1^). However, their collisions with the seabed crush and recycle vast benthic communities, costing an estimated 4 × 10^4^ t C yr^−1^. We calculate that giant iceberg formation (ice shelf disintegration) has a net potential of approximately 10^6^ t C yr^−1^ sequestration benefits as well as more widely known negative impacts.

This article is part of the theme issue ‘The marine system of the West Antarctic Peninsula: status and strategy for progress in a region of rapid change’.

## 1. Introduction

The strongest measured and most visible impact of climate change in the polar regions has been the drastic shift in sea-ice extent. Over the last four decades, the seasonal sea-ice maximum area and duration have decreased considerably over the Arctic and West Antarctica [1]. By contrast, around parts of East Antarctica, such as the Weddell and Ross Seas, there have been ever stronger sea-ice gains [2]. Alongside this, there have been other important marine-ice changes, including increases in the number of marine glaciers in retreat and their retreat rate, as well as major ice shelf disintegrations [3]. Reductions in West Antarctic sea ice, particularly the winter fast ice (here defined as the seasonal frozen sea surface, which is connected to land), have already seen pronounced responses from pagophilic (ice-associated) higher predators and the highly productive pelagic zooplankton, such as Antarctic krill [4]. In our work, we focus on marine-ice interactions with life on the Southern Ocean seabed (benthos) for several reasons: (i) it is less well known and understood; (ii) the vast majority of Antarctica's species live there and nearly all are endemic (live only there); and perhaps most importantly (iii) benthos perform a key ecosystem service of carbon storage and burial, known as blue carbon [5]. Blue carbon (carbon in organisms) in polar waters has been little considered to date, with scientific attention mainly focusing at lower latitudes on kelp forests, seagrass beds, salt marshes and mangrove swamps (which are contracting due to anthropogenic habitat use, pollution and other disturbance). However, benthic blue carbon around Antarctica has been found to strongly interact with sea-ice losses to make it a globally significant negative (mitigating) feedback on climate change. Indeed, there is recent evidence that polar benthic blue carbon stocks, and thus their potential for mitigating impact, have increased [6]. The biggest factor limiting build-up, longevity and blue carbon storage potential of benthic communities is the scouring of icebergs (originating from glaciers or ice shelves) when they run aground on the seabed [7]. This smashes up and grinds carbonate bioconstruction, providing feasts for scavengers [8] and reducing burial, but scour decreases rapidly in frequency with depth [9]. While being locally catastrophic, iceberg scour promotes regional biodiversity by making the seabed a patchwork, with each location at a varying state of recovery since it was last scoured, across the Arctic [10] and Antarctic [7] continental shelves. While studying iceberg frequency and impact is easiest on the shore and in the shallows [11,12], giant icebergs, several kilometres in length, are big enough to effectively track using remote sensing. In this paper, we provide evidence to support the inclusion of (fast ice and) icebergs as an important component of a holistic Southern Ocean Observing System (SOOS) because, apart from marine traffic and installation safety, icebergs have a profound impact on blue carbon ecosystem services.

Periodic disintegration of polar ice shelves to form giant icebergs is a normal process during warmer interglacial phases, such as the current time. However, seabed scour patterns suggest that the most recent past deglaciation mainly formed smaller icebergs (with V-shaped keels) than current ice shelf losses, which are producing giant tabular icebergs [13]. The rapidity and extent of recent ice shelf collapses into giant icebergs has been increasingly linked to anthropogenic enhancement of climate change through fossil fuel use. Giant icebergs (greater than 30 km^2^) are not uncommon, currently numbering 47 larger than 30 km^2^ in March 2017, including six that exceeded 1000 km^2^ in area (see US National Ice Center iceberg data available at http://www.polarview.aq/antarctic). Duprat *et al.* [14] were able to study some effects of 17 recently calved giants in detail and begin a viewpoint of positive climate feedback contributions. Ice shelf fragility has been recently highlighted by the recent giant iceberg (A68) breakout of Larsen C and the long, rapidly growing fissure in the Brunt ice shelves in the East Antarctic—the latter forcing temporary evacuation of the Halley VI UK Antarctic research station. The consequences of such calving can be severe, including changing albedo (and thus heat absorption) and increasing the seaward flow of land-based ice by up to a factor of 8, at least initially [15]. The change in macro-coastline and the (indirect) potential for sea-level rise consequences of ice shelf losses have gathered considerable public and scientific attention, particularly since the collapses of more northerly parts of Larsen in 1995 and 2002. Ice shelf disintegrations and calving have formed icebergs up to 32 000 km^2^, the passage and fate of which have also captured widespread concern, from collisions with rich life on the seabed or anthropogenic installations (such as oil rigs [16]) to indirect impacts on penguin mortality [17].

The positive feedback on climate change and potential for serious societal impacts of increased ice shelf losses are considerable but these are by no means the only important effects. The continental shelves part-covered by ice shelves are biologically unproductive but their collapse may open up vast new bays to generate new primary productivity [18], secondary productivity [19] and thus major benthic carbon capture and storage opportunities. The giant icebergs fertilize the otherwise poorly productive ocean away from the shelf as they drift northwards, likewise generating new primary and secondary productivity, which if converted into long-term blue carbon stores will produce a negative feedback on climate change.

Here we consider recent progress in measuring and understanding more general relationships between sea ice, icebergs and blue carbon on the Southern Ocean's seabed. Our aim is to better understand what we know and do not know about sea ice and iceberg impacts on blue carbon around the Southern Ocean. From published papers we analysed recent (2014–2017) benthic blue carbon data from (i) the relationship between fast-ice duration and the frequency of ice scouring in shallow waters at Rothera and Carlini stations on the West Antarctic Peninsula (WAP), (ii) iceberg impacted and non-impacted sites on South Georgia's north shelf and (iii) how correlation changes with depth between sea ice and benthic blue carbon around Marguerite Bay. Fourthly, we also analysed US National Ice Center circumpolar data of giant iceberg status (e.g. fasted, grounded and roaming), grounding hotspots and tracks to assess the trade-offs of giant iceberg formation with respect to effects on benthic ecosystem services of blue carbon (e.g. enhanced growth; carbon storage and its fate; potential for sequestration). Finally, we used these findings and literature to construct a polar projection map (based on 3° × 3° grid cells) of data illustrating benthic blue carbon change where we completely lack data.

## 2. Fast ice, icebergs and benthic blue carbon

**Figure 1.**
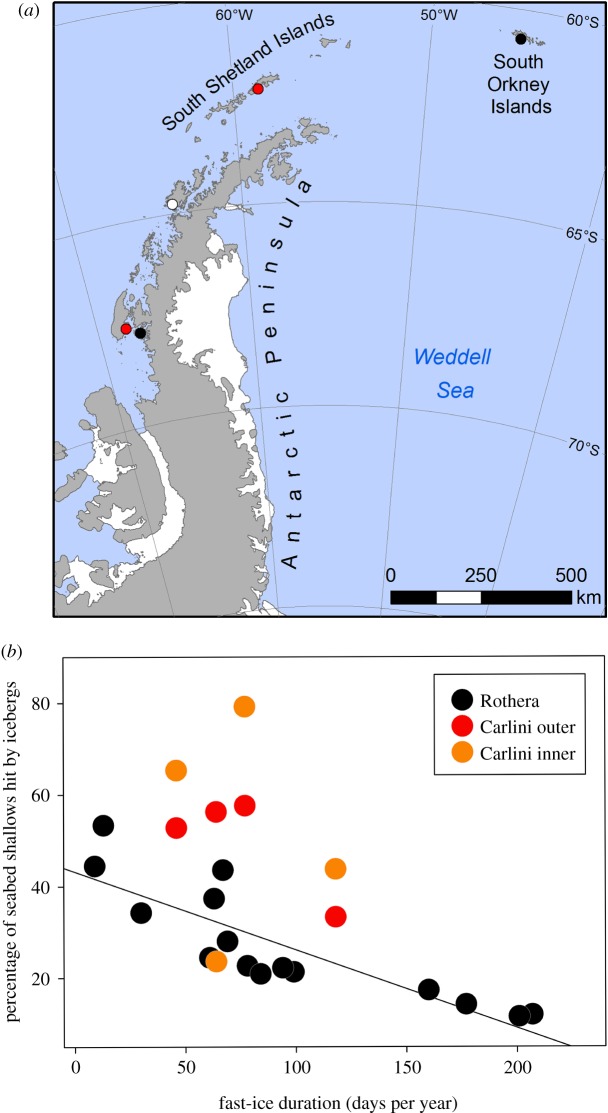
Monitoring sea ice and iceberg activity in West Antarctica. (*a*) Monitoring sites, using automated cameras (black circles) and manual observations (white circle), of sea-ice extent in time and space. Monitoring sites of both sea-ice extent and annual iceberg scouring (red circles) at Rothera (bottom red circle) and Carlini (top red circle) [9,21]. (*b*) Relationship between duration of fast-ice cover each year and ice scouring on the seabed at Rothera and Carlini (inner bay in orange, outer bay in red) stations. The *y*-axis shows the proportion of the monitored seabed from a 5–25 m depth hit by icebergs. Pearson's correlation line (−0.86, *p* < 0.001) drawn through Rothera data only.

**Figure 2.**
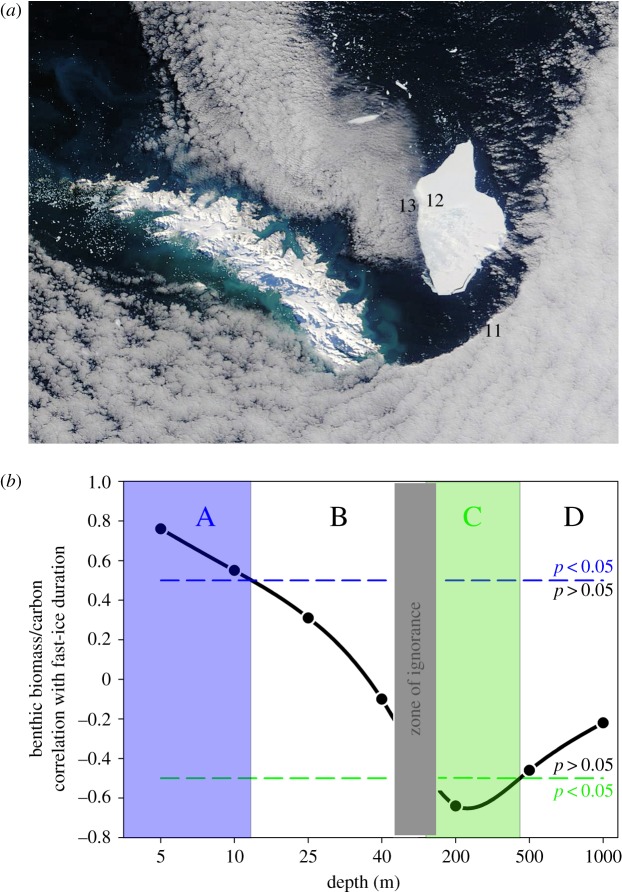
Iceberg impacts on seabed blue carbon. (*a*) Iceberg grounded at South Georgia in 2004; photo courtesy of US National Ice Center. Sites 11, 12 and 13 are benthic image and trawl samples. The island in the image (left) is South Georgia and iceberg size is 75 × 41 km. (*b*) Correlation between sea-ice duration and blue carbon storage on the seabed around southern Adelaide Island, West Antarctic Peninsula (from data in Barnes [6,9] ). Zones are zoobenthic carbon; significant increase with increased fast-ice duration (A), no significant change (B,D), significant decrease with increased fast-ice duration (C) and depth zone where there is too little known for meaningful analysis (zone of ignorance).

The Southern Ocean sea surface features striking contrasts in albedo between open water and sea ice, and as such remote sensing is an ideal tool to detect trends in sea-ice spatial and temporal extent [2]. Satellite data can be enhanced (when cloud obscures satellite image capture) and ground-truthed by *in situ* manually operated or remote image capture. Around Antarctica these have proved to be complex, in terms of extent, geography and drivers of change. Overall, there have been marginal increases in Southern Ocean sea ice to date, but there have also been considerable losses around West Antarctic continental shelves and even larger gains, mainly over deeper water, in the Weddell and Ross Seas [1]. The consequences of sea-ice losses and gains remain poorly understood but strongly influence local physical characteristics such as heat and gas exchange, biological activity such as phytoplankton bloom timing, duration and composition [20,21], and iceberg scouring rates [9,22]. Research has identified diverse and far-reaching secondary consequences to fauna such as habitat and food provision, impacting breeding of higher predators to feeding and composition of zooplankton [23,24]. Near-coast sea-ice losses and responses of benthic organisms to such sea-ice losses are hard to monitor as station-based direct observation is crucial [6,9,25]. Such work is both time- and labour-intensive and only yields small spatial coverage (rarely more than square kilometres), so collaborative efforts are needed to increase coverage and analyse resulting data [26]. To date few locations maintain ‘long-term’ observations of sea-ice status (figure 1*a*) to ground-truth coastal remote-sensed data, and even fewer are monitoring benthos responses (red circles, figure 1*a*). Circumpolar satellite-derived data have revealed that the WAP particularly is a hotspot for sea-ice losses. Iceberg scour can be very frequent in the shallows if the area above it is free of fast ice (because there is less obstruction to wind-driven travel) [9,22]. New data from seabed-ice scour monitoring adjacent to Carlini station [25] (figure 1*b*) shows even higher rates than at Rothera. As at Rothera, the site (at Carlini) closest to a glacier terminus showed some very high scouring rates, suggesting that local iceberg production as well as fast ice are important factors for seabed disturbance. We suggest that climate forcing has made the WAP a hotspot for coastal iceberg scouring. Results from the longest running (2003 onwards) iceberg scour-monitoring experiment near Rothera research station [9] showed that ice scouring can reduce by half the carbon held by shallow benthos in a single year. Scaled up to the area of the WAP shallows, this may annually recycle an estimated 8 × 10^4^ tonnes of carbon per year [9] that otherwise could have been buried—and represents a major source of previously unaccounted variability in carbon capture and storage. That study showed that blue carbon standing stock recovery took 4–6 years in the shallows. This is hard to test at typical shelf depths but we reanalysed 36 samples of benthic images with matched trawl specimens from Barnes & Sands [26] collected in 2011 from site 12 (at which Polarview showed an iceberg was grounded in 2004, figure 2*a*), and sites 11 and 13 (which showed no evidence of having been scoured in the remote-sensing era). We found no significant difference between scoured and unscoured samples with respect to blue carbon stock mass (ANOVA, df1, *F* = 1.69, *p* = 0.2), suggesting recovery is at least possible within approximately 7 years even at a 200 m depth on the South Georgia shelf. Part of the problem and complexity of analyses of iceberg blue carbon impact, at least coastally, is the confounding effects of sea ice and phytoplankton blooms. In shallow coastal waters, sea-ice losses drive both blue carbon decreases by increased scour probability, and increases through longer phytoplankton blooms sustaining benthic growth. To examine the relationship between sea ice and benthic biomass, we reanalysed data presented in Barnes [6,9] from Marguerite Bay (1997–2016) and examined the relationship between benthic biomass (blue carbon storage) and fast-ice duration, and how this changes with depth from the shallows down to 1000 m (data from figure 2*b*). We found that, in the top approximately 10 m, there was a positive correlation between blue carbon and sea-ice duration (zone A, figure 2*b*). We interpret this as longer sea-ice durations reduce ice scour chances, which outweighs slower growth from the shorter phytoplankton blooms. There was no relationship between benthic blue carbon and sea-ice duration from 10–40 m depth (zone B, figure 2*b*), which is probably explained by the reduction in ice scour chances due to longer sea-ice durations approximately balancing slower growth from the shorter phytoplankton blooms. At typical continental shelf depths (200–500 m), there were negative correlations between blue carbon and sea-ice duration (zone C, figure 2*b*). Ice scour chances are so rare in deep water (because few icebergs are big enough to have keels hundreds of metres deep) that longer sea-ice durations have little impact on this, so the main effect is slower growth from shorter phytoplankton blooms. Between 40 and 200 m is a zone of very little benthic sampling, perhaps often too shallow or geographically close to shallows for safe ship operation but too deep for self-contained underwater breathing apparatus (SCUBA)—a data-poor zone that we need to address (figure 2*b*).

## 3. Blue carbon trade-offs of giant icebergs

### (a) How important are the positive blue carbon effects of giant iceberg formation?

Away from the shallows, much larger icebergs (by area) are easier to track from genesis to eventual exit from the Southern Ocean, yet much less is known of the subsurface impacts of these giants on blue carbon, although recent studies have highlighted their importance in their pelagic fertilization roles. Here, we discuss and quantitatively estimate the importance of blue carbon impacts of Southern Ocean icebergs.

**Figure 3.**
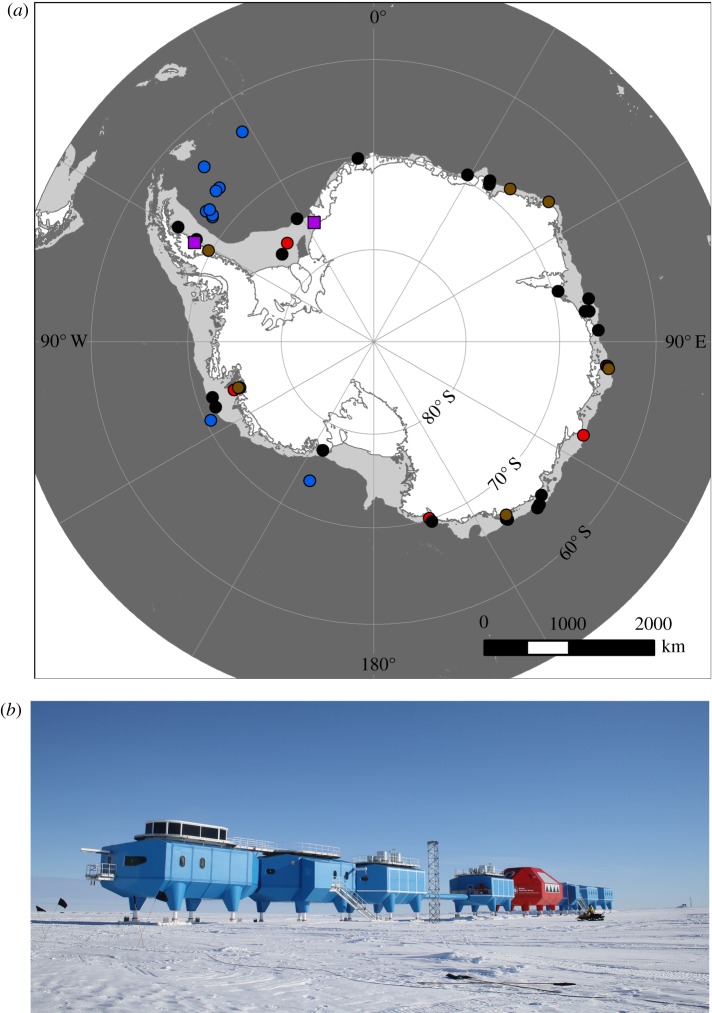
Giant icebergs in the Southern Ocean. (*a*) Distribution of calved (circles) and calving (squares) giant icebergs (greater than 30 km^2^) around Antarctica, in which the symbols are giant icebergs. The calved icebergs are further categorized as grounded (red), roaming free on the continental shelf (black) or in deep water (blue), or stuck in fast ice (dark purple). (*b*) Is the UK's Halley VI research station set to become the first giant-iceberg-based research station?

Giant icebergs mainly originate from ice shelf disintegration and can remain fasted in sea ice for years in deep coastal water, roam on or off the continental shelf or run aground (figure 3*a*). Giant icebergs leave a trail of trace nutrient enrichment, enhancing phytoplankton blooms in their path [14]. This phytoplankton bloom fertilization was found to increase bloom intensity by an order of magnitude [14], and increase the potential blue carbon capture, but whether this increases any storage or sequestration by benthic organisms depends on where and when this occurs. Giant icebergs could travel over hundreds of kilometres of productive continental shelf seabed. For example, Brunt ice shelf-originating giant icebergs could drift southwest and then north with prevailing winds and currents across the huge shelf area of the Southern Weddell Sea [27]. Beyond the continental shelf, icebergs travel over much more sparsely populated abyssal fauna, where bloom fertilization is less likely to have significant impact on (benthic) storage or sequestration. However, if giant icebergs are calved during the summer and intercept phytoplankton blooms, increased intensity of those blooms is unlikely to greatly increase blue carbon capture or storage, as there is no evidence that planktotrophic shelf benthos are food-limited during summer bloom periods [6,9]. Significant lasting impact of giant iceberg bloom fertilization (in terms of enhanced blue carbon storage—as opposed to carbon cycling) is likely to be most associated with icebergs calved in spring or autumn (because phytoplankton abundance in summer exceeds animal capacity to eat) and those with some distance to travel before reaching the continental shelf break. Although carbon cycling increases driven by giant iceberg formation have been estimated [14], contributions to benthic blue carbon storage are not straightforward, despite probable importance—and would need to be calculated on an ‘iceberg by iceberg’ basis.

Giant icebergs have another very important and different impact on enhancing blue carbon storage—their calving involves break-off from an ice shelf. As in current time all ice shelves occur over continental shelf, their break-up to form giant icebergs creates new areas of open water above potentially highly productive seabed. In the last few decades more than 10^4^ km^2^ of ice shelf have collapsed, to open up continental shelf waters to primary production and secondary benthic carbon capture and storage [18]. New benthic production in such an area was estimated to be nearly 10^6^ tonnes of carbon per year (of which nearly half could be stored (immobilized) within the skeletal matrix of the benthos) [6]. If the Peck *et al*. [18] estimates are approximately correct, the new open water left by calving the new 5000 km^2^ iceberg from Larsen C could generate 7 × 10^4^ tonnes of C^immobilized^ per year (even without consideration of additional fertilization effects). However, four years after the Larsen A and B ice shelf collapses, Fillinger *et al*. [19] reported ‘two- to threefold increases' in benthic sponge density and biomass (and that sponges were the dominant component of the fauna). This suggests that the Peck *et al*. [18] estimates based on steady-state increases may be conservative. Even if only for the first few years of new colonization and productivity, newly exposed shelf areas clearly capture and store carbon more rapidly than ‘normal’ annual incremental growth. Thus, a new 5000 km^2^ iceberg creation may actually generate more than 10^5^ tonnes of C^immobilized^ per year (calculation is Peck *et al*. [18] estimate × Fillinger *et al*. [19] growth rate = 7 × 10^4^ × 2.5 = 2 × 10^5^). Such productivity could be increased because of (a) additional climate-forced sea-ice losses in some regions (e.g. Antarctic Peninsula) [5,6], and (b) giant iceberg phytoplankton bloom fertilization along its track over continental shelf [14]. The magnitude of such (a,b) increases could be ×2 for sea-ice losses [5] and ×5–10 for giant iceberg fertilization [14]. Thus we estimate that the 2 × 10^5^ tonnes of C^immobilized^ per year estimate is more likely to be approximately 10^6^ tonnes of C^immobilized^ per year (calculation is Peck *et al*. [18] estimate × Fillinger *et al*. [19] growth rate × Duprat *et al*. [14] iceberg fertilization = 7 × 10^4 ^× 2.5 × 5 = 10^6^, for each 5000 km^2^ iceberg) as a conservative negative feedback consequence of giant iceberg formation along the Antarctic Peninsula area of the polar regions alone.

### (b) What are the offset negative blue carbon effects of giant iceberg formation?

Small icebergs can halve incremental growth of benthic communities and thus the potential for carbon storage, as happened between 2007 and 2009 in Ryder Bay near Rothera research station (WAP) due to ice scour [9]. The creation of giant icebergs has many other effects besides those which can be considered negative feedbacks on climate change, not least including threats to installations (figure 3*b*). When ice shelves collapse to form giant icebergs, the buttressing of land-based ice sheets is reduced [28,29]. Thus calving typically results in acceleration of land-based ice towards the sea, driving sea-level increases from any newly marine-based ice. This does not have an obvious direct positive or negative consequence on blue carbon storage but can accelerate deglaciation. New open water areas result in a strong albedo (reflectivity) change—from white to blue. This is a positive feedback on climate change because of the resulting extra heat absorbed. Depending on the extent of warming, this could have minor effects on benthos blue carbon capture: an increase in growth due to increasing meal processing rate [6,9] or a reduction of growth when temperatures proceed beyond organisms’ thermal tolerance envelopes [30]. More open water also has increased potential for gas exchange and thus CO_2_ absorption, which could reduce benthic carbon immobilization and sequestration through ocean acidification [31]. This occurs not only because of increases in the physiological ‘cost’ of synthesizing and maintaining carbonate skeletal material but also because of the greater chance of it dissolving (after death) before burial.

**Figure 4.**
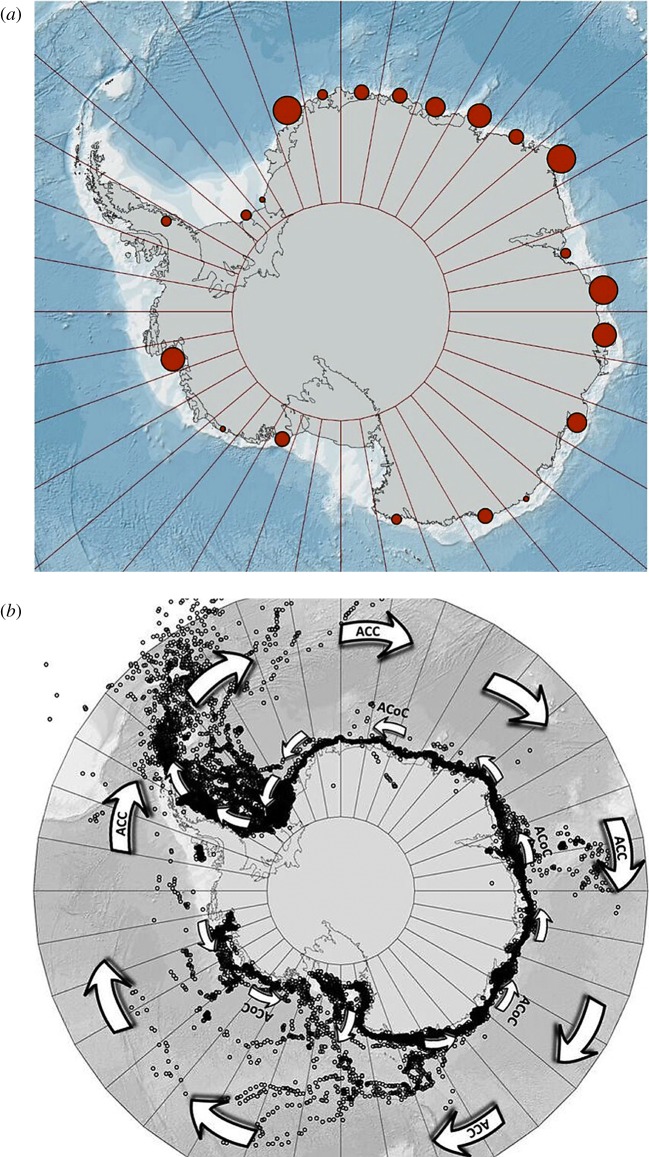
Hotspots and tracks of giant icebergs. (*a*) Hotspots of giant icebergs grounding on continental shelf around Antarctica from 2014 to 2017 (see http://www.polarview.aq/antarctic for data). (*b*) Historic tracks of giant icebergs, together with the major current systems (Antarctic Circumpolar Current (ACC, large arrows) and Antarctic Counter Current (ACoC, small arrows)) influencing these tracks. Historic iceberg information provided by US National Ice Center from Southern Ocean iceberg tracking database (see http://www.natice.noaa.gov/doc/Notice_Iceberg_Tracking_Criteria.pdf).

The major negative impact of giant icebergs on benthic blue carbon is if they collide with the seabed and scour. Giant icebergs would only ground and scour in deeper water, which may on average only occur once every few hundred years at any one location [7]. However, the residence time of some, e.g. A23 and B12, can be years to decades, which makes scour highly probable for the largest icebergs. For example, two of the largest three icebergs currently around Antarctica (A23A (1760 km^2^) and B22A (1056 km^2^)) were grounded in 2016/2017 in the shelf seabed. Such grounding can be a prolonged occurrence; for example, A23A has been grounded for more than three decades, since 1986. The chances and location of any particular iceberg scouring depend not only on size (most 2016/2017 giants were 100–500 km^2^ in area) but also on shape, calving location and current and wind direction and strength, as well as stochastic factors such as which eddies entrain it. Larger icebergs can be tracked such that their motion and fate can be followed; for example, in early 2017 there were four icebergs (8.5%) larger than 30 km^2^ in area grounded, and thus scouring blue carbon. The extent of this could be ground-truthed using ship-based multibeam, swath bathymetry data collection. Benthic imaging and sampling could then quantify blue carbon losses and, as such scours can be many kilometres long, they are likely to crush and recycle hundreds–thousands of tonnes of blue carbon standing stock. For example, benthos in the vicinity of the grounded scouring giant icebergs A23A and B22A hold 8–16 tonnes of standing stock of carbon per square kilometre [5,32]. Imagery and sampling in new deep water scours show near-complete destruction of fauna [7,10]. Many such scours occur in iceberg hotspots, which reduces blue carbon losses (repeat scouring by an iceberg of a site in an early recovery stage recycles less carbon than scouring of a rarely hit area of climax benthic fauna). Scouring climax benthic communities might recycle 16 tonnes of carbon per square kilometre (the maximum standing stocks of benthos from [5,32]). We found that there were clear hot- and coldspots of iceberg scouring in deep waters around Antarctica (figure 4*a*). The Lazarev, Cosmonauts, Davis and East Amundsen Seas were hotspots of deep shelf grounding, whereas there was little deep-water scouring in the Bellingshausen and Ross Seas. The paths of giant icebergs (from US National Ice Center satellite data) also showed distinct patterns, firstly following the anticlockwise coastal current, then leaving the shelf from the Eastern Weddell and Ross Seas and being dispersed by the Antarctic Circumpolar Current (figure 4*b*).

Even if only one-fifth of giant icebergs of the size of A23A/B22A were grounded, more than 5 × 10^3^ tonnes of benthic carbon per year could be recycled rather than stored and buried. To date, such losses have not been quantified in absolute frequencies at depths relevant to giant icebergs. Even scouring has some positive blue carbon aspects in terms of potential to bury benthos in sediment berms, resuspend food material (enhancing growth in nearby benthos) and open up space for faster-growing benthic pioneer species [33].

### (c) Net effect of giant icebergs on blue carbon ecosystem services

Interglacial phases have probably seen major fluctuations in continental shelf capture and stores of blue carbon by zoobenthos in the polar regions with ice shelf collapses and the impact of the resulting giant icebergs [34]. Overall, this drives a net gain of blue carbon, and importantly this extends to a net gain on the seabed where long-term storage chances and sequestration are maximal. However, for any one particular iceberg, the blue carbon gain may be small or even a net loss if it scours shelf benthos, depending on the size of the scoured area. Even then, the net gain should be positive over decades, unless the scoured area is bigger than the area of the iceberg. Scour size and scouring probability by giant icebergs, both of which are unknown, are important values. Two new giant icebergs of 5000 km^2^ would typically be expected to generate 2 × 10^6^ tonnes of C^immobilized^ per year in total (estimate from §3a (×2 for two icebergs)) and ‘cost’ up to 16 tonnes for each km^2^ scoured (estimate from §3b) [5,32] at a Weddell Sea locality (where 33% of giant icebergs were in 2016/2017). In the Amundsen Sea generation of such icebergs would be expected to generate less than half this blue carbon but similarly ‘cost’ half as much through scouring. The part of the equation missing from estimating a net carbon effect of giant icebergs is how much area they scour. This is unknown, but based on US National Ice Center tracking, the estimate used here is that eight giant icebergs ground per year. Observations in Ryder Bay (WAP) of grounded icebergs suggest that up to a quarter of the area of each grounded iceberg scours. So ‘costs’ are 0.25 (maximum proportion of iceberg grounded)×total estimated area of grounded giant icebergs (10 000) × 16 tonnes (stored carbon in benthos covering each km^2^ which becomes recycled) = up to 4 × 10^4^ tonnes of C^immobilized^. This cost is likely to be highly overstated because (a) the largest giants can be grounded in the same location for many years (but only recycle benthic carbon initially) and (b) it assumes icebergs become grounded at random locations on the continental shelf. It is more likely that there will be hotspots of scour on rises or sills, such as the continental shelf break, which will be hit more often and have less time to recover (and thus less stored carbon is recycled). We calculate that the net carbon gain of giant icebergs is at least 2 × 10^6^ − 4 × 10^4^ = 1.9 × 10^6^ tonnes of C^immobilized^ per year. This is equivalent of the carbon exhaust output of 400 000 cars per year, or nearly four million cars in terms of total zoobenthic carbon (compared with the smaller proportion of carbon which is immobilized [5]). This is equivalent to or more than all the car outputs in any one of up to two-thirds of the world's countries.

Thus, there is a strong argument that giant iceberg formation has a net positive climate benefit by growing important negative feedbacks [14,18]. Of course cost–benefit analysis of giant icebergs concerns much more than blue carbon, and recovery to previous climax biodiversity may take many decades to hundreds of years [7,10]. Weighing up potential blue carbon gains versus buttressing of ice sheet losses, sea level, gas exchange and heat absorption changes would be a complex calculation, involving some stochastic and chaotic elements. Such a calculation, if meaningfully possible, might not support the positive viewpoint of ice shelf disintegration, especially if there was a very high-profile and expensive research station on one of them.

### (d) Wider context of importance of biological production to Antarctic carbon sequestration

Zoobenthic production around Antarctic continental shelves varies considerably with geography and depth, but can be approximately 13 g C m^−2^ yr^−1^ in the shallows [6], through to approximately 5 g C m^−2^ yr^−1^ at 100–300 m and approximately 1.5 g C m^−2^ yr^−1^ below 300 m depth [32,35]. Production in the upper 100 m is little studied and complicated by ice scour, but at least along the WAP has been estimated as equalling the total (continental shelf) below it [6,9]. Most data occur for 100 m and below, so an estimate of Antarctic zoobenthic production was made from this [32,35]. If an approximate mean of organic carbon of 3 g C m^−2^ yr^−1^ is used, scaled up to 3 t C km^−2^ yr^−1^ and the same amount of carbonate (which varies enormously and of which only 12% is carbon), this gives a total of approximately 3.4 t C km^−2^ yr^−1^. If the shallows below 100 m are of similar value, then 3.4 × 2 = 6.8 t C km^−2^ yr^−1^. Multiplied by the area of Antarctica's continental shelf (4.4 × 10^6^ km^2^), it is approximately 30 Mt C yr^−1^. This figure is considerably less than 1% of annual anthropogenic emission; however, production has doubled (at least in some biota) since the 1990s around many Antarctic shelves due to sea-ice changes [5,6], so this estimate may now be closer to 60 Mt C yr^−1^. This has to be further increased to account for ice shelf losses opening up large new areas of benthic production, estimated to generate as much as 10 Mt C yr^−1^ [18]. Yet such newly ice shelf-free areas have been found to be two to three times more productive [19] than originally thought [18]. The current work estimates that we need to add a million tonnes for each 5000 km^2^ iceberg calved. Each of these increases to the initial estimate adds considerable uncertainty, but may be approximately 80 Mt C yr^−1^ (initial estimate at 30 Mt C yr^−1 ^× 2 for increases due to sea-ice losses [5,6] (+10 Mt C yr^−1^ for new production with ice shelf losses [18] × 2 for new estimates of growth rates in new ice shelf loss areas [19])). This approximate 80 Mt C does not take into account that there is as much continental shelf at sub-Antarctic latitudes and that many of Antarctica's outlying islands [5] and sub-Antarctic islands can be much more productive [36] than those further south (with exceptions [26]). Doubling our estimate to approximately 160 Mt C yr^−1^ to account for sub-Antarctic continental shelf areas, while appearing large, is only marginally more than 1% of global anthropogenic carbon output. We would argue that it is more important than its empiric value suggests because benthos is at the principal sites of burial, with genuine sequestration potential. This is unlike more considerable but more uncertain values dissolved in the ocean (due to temporal or spatial outgassing) or held in pelagic or forest biomass, which has much more time and space to be broken down on death and recycled in the microbial loop. Increases in sea temperature could yet increase zoobenthic carbon storage further [37], but this may be more than counteracted by ocean acidification, adding yet more uncertainty to estimated totals. The current work aimed to estimate the iceberg component of biological carbon sequestration in the southern polar region. Thus, our contextual calculations of total southern polar region zoobenthic carbon storage is only approximate, with considerable error, but it does suggest that icebergs have an important role (10–20%) within this biological component, as recently suggested [14].

## 4. Conclusion

Thus we conclude that sea ice interacts strongly with benthic blue carbon ecosystem services, partly through iceberg activity.

**Figure 5.**
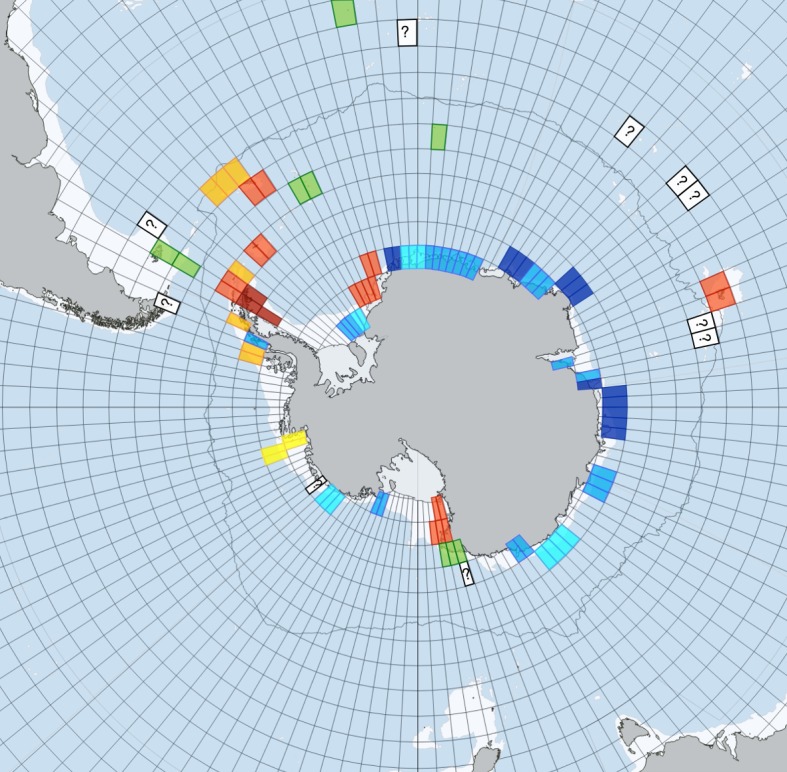
Trends in benthic blue carbon change. Change in blue carbon benthic standing stock (tonnes per square kilometre) around the Southern Ocean. Each cell is 3° × 3°. Cell data based on: (i) yellow–red are increases from direct (increased standing stock from sampling benthos) and indirect (iceberg grounding data) evidence streams, Fillinger *et al*. [19], Barnes [5,9], Barnes & Sands [9]; (ii) white with question mark are samples awaiting analysis (from Antarctic Circumnavigation Expedition cruise 2016–2017) and expected samples from British Antarctic Survey JR17004 scientific cruise; (iii) light to dark blue are decreases from giant iceberg grounding probabilities given by US National Ice Center (figure 4*a*).

It is clear that sea ice and icebergs have complex influences on benthos and their blue carbon ecosystem services—and thus on the power of feedbacks on climate change. Pooling literature data sources [5,6,9,14,15,19,26,27,32] and iceberg-grounding hotspots in the current paper, we constructed a map of change in benthic blue carbon storage ecosystem services. Unsurprisingly, we found no data for even the majority of continental shelves around Antarctica, but we highlight likely blue carbon losses (blue cells, figure 5) in regions of frequent giant iceberg grounding. Fillinger *et al*. [19] have shown large blue carbon gains (albeit reported as colonization and growth of sponge increases) around collapsing ice shelves (dark red, figure 5). Thus, the recent declaration that such areas attain Marine Protected Area status is good news for conservation of strong blue carbon ecosystem services, especially as evidence suggests these will strengthen if climate forces sea-ice losses [6]. Benthic blue carbon storage has increased at varying levels (yellow–red, figure 5) around West Antarctic shelves, including parts of the Weddell and Ross Seas [5]. The recent international Antarctic Circumnavigation Expedition (ACE) enabled various samples to be taken at key locations (white cells with ‘?’, figure 5), which should reveal blue carbon trends following analysis. Finally, there are areas for which there are some samples but for which no trend is apparent (green, figure 5), though this may be from paucity of samples, analyses or unclear identification.

Our calculations suggest that giant icebergs alone contribute a net gain of 10^6^ tonnes of C^immobilized^ per year (around the Antarctic Peninsula), which matches similarly sized gains in the same region driven by sea-ice losses [9]. The considerable interest and concern about icebergs, particularly giants, focuses on potential natural environment ‘damage’. However, our work supports recent studies (e.g. [19]), which show that iceberg effects are more complex and can aid ecosystem development and identification of negative feedbacks on climate change.

On the scale of terrestrial forests and oceanic water absorption, Antarctic benthos carbon cycling is small, at just 10^6^ tonnes for increases from ice shelf losses (by our calculations) in addition to 10^7^ tonnes for increases driven by sea-ice losses [7,9]. Recently, Ashton *et al*. [37] demonstrated that in Antarctic coastal waters warming of just one degree increases growth by more than expected amounts. This gives reason to hypothesize that benthos on the warmer and vast Arctic and sub-Antarctic shelves (e.g. 2.2 × 10^6^ km^2^ of Kerguelen Plateau) may also be increasing blue carbon storage—and when quantified could prove the single largest negative feedback (on climate change). All three (Antarctic, sub-Antarctic and Arctic) of these sinks are expected to increase in response to climate change, in contrast to shrinking forests (and in the sea mangroves, seagrass beds and saltmarsh areas) and become progressively less efficient at ocean sponging [38]. Sub-Arctic forests do represent a negative feedback [39] but of smaller magnitude than polar and subpolar continental shelves. Cold water benthic blue carbon responses to (Arctic and West Antarctic) losses of sea ice, ice shelves and warming probably represent the three largest negative feedbacks yet among those which are most poorly characterized and understood. The current work at least attempts to evaluate the iceberg component of these.

## Data accessibility

This article has no additional data.
